# Multigene Assessment of the Species Boundaries and Sexual Status of the Basidiomycetous Yeasts *Cryptococcus flavescens* and *C*. *terrestris* (Tremellales)

**DOI:** 10.1371/journal.pone.0120400

**Published:** 2015-03-26

**Authors:** Andrey Yurkov, Marco A. Guerreiro, Lav Sharma, Cláudia Carvalho, Álvaro Fonseca

**Affiliations:** 1 Leibniz Institute DSMZ-German Collection of Microorganisms and Cell Cultures, Brunswick, Germany; 2 Centro de Recursos Microbiológicos, Departamento de Ciências da Vida, Faculdade de Ciências e Tecnologia, Universidade Nova de Lisboa, Caparica, Portugal; University of Minnesota, UNITED STATES

## Abstract

*Cryptococcus flavescens* and *C*. *terrestris* are phenotypically indistinguishable sister species that belong to the order Tremellales (Tremellomycetes, Basidiomycota) and which may be mistaken for *C*. *laurentii* based on phenotype. Phylogenetic separation between *C*. *flavescens* and *C*. *terrestris* was based on rDNA sequence analyses, but very little is known on their intraspecific genetic variability or propensity for sexual reproduction. We studied 59 strains from different substrates and geographic locations, and used a multilocus sequencing (MLS) approach complemented with the sequencing of mating type (*MAT*) genes to assess genetic variation and reexamine the boundaries of the two species, as well as their sexual status. The following five loci were chosen for MLS: the rDNA ITS-LSU region, the rDNA IGS1 spacer, and fragments of the genes encoding the largest subunit of RNA polymerase II (*RPB1*), the translation elongation factor 1 alpha (*TEF1*) and the p21-activated protein kinase (*STE20*). Phylogenetic network analyses confirmed the genetic separation of the two species and revealed two additional cryptic species, for which the names *Cryptococcus baii* and *C*. *ruineniae* are proposed. Further analyses of the data revealed a high degree of genetic heterogeneity within *C*. *flavescens* as well as evidence for recombination between lineages detected for this species. Strains of *C*. *terrestris* displayed higher levels of similarity in all analysed genes and appear to make up a single recombining group. The two *MAT* genes (*STE3* and *SXI1/SXI2*) sequenced for *C*. *flavescens* strains confirmed the potential for sexual reproduction and suggest the presence of a tetrapolar mating system with a biallelic pheromone/receptor locus and a multiallelic HD locus. In *C*. *terrestris* we could only sequence *STE3*, which revealed a biallelic P/R locus. In spite of the strong evidence for sexual recombination in the two species, attempts at mating compatible strains of both species on culture media were unsuccessful.

## Introduction

Knowledge on the reproductive mode of fungi is central to understanding their life cycles and ecological strategies. The ratio of clonal expansion and meiotic recombination within a fungal species determines its population structure and boundaries, as well as its adaptive potential. The capacity for sexual recombination in fungi can be assessed by laboratorial testing for mating ability, but this strategy is often technically cumbersome or not viable in species that require complex conditions to develop the sexual structures, such as strict host associations. Alternatively, it is possible to search for functional mating type (*MAT*) loci, i.e., the sex-determining regions of the fungal genome, e.g. [1]. This approach has revealed different mating compatibility systems in basidiomycete fungi (bipolar, tetrapolar or pseudobipolar) and has provided clues to explain differences in sexual behavior (heterothallism, homothallism or same sex mating) [2]. Another indirect strategy to assess the reproduction mode in fungi consists in using molecular population genetics based on multilocus sequence analyses [3–4]. Studies relying on the latter approach have revealed that many putatively asexual fungal taxa are in fact mosaics of recombining and clonal populations, hinting to the existence of cryptic sexual cycles and suggesting that exclusively asexual taxa are evolutionary endpoints, e.g. [3]. Most of those studies have focused on human or plant pathogens with less attention given to those fungi that lack a more obvious applied impact.

In depth information on population structure and reproductive mode of basidiomycetous yeasts of the genus *Cryptococcus* is at present available only for the human pathogens *C*. *neoformans* and *C*. *gattii*, e.g. [5], which belong to the order Tremellales. Many other species of this polyphyletic genus are saprobic and are found consistently in different natural environments such as plant surfaces or soil [6–7], but very little is known on their intraspecific genetic variability or population structure. *Cryptococcus* species within the Tremellales are putatively asexual, but are phylogenetically intermingled with members of sexual genera (e.g. *Tremella*) and may represent yeast states of unrecognised sexual taxa [6, 8–9]. Studies involving molecular typing of large collections of strains of *C*. *neoformans* and *C*. *gattii* have demonstrated the coexistence of both recombination and clonal expansion, the likely presence of further cryptic species, as well as the occurrence of same sex mating and of inter-specific mating [5, 10]. However, no other species of *Cryptococcus* have been studied in such detail regarding their population structure and mode of reproduction.

The mating behavior and *MAT* loci structure has been elucidated for some yeast species belonging to the Tremellales [11–12]. *C*. *neoformans* and *C*. *gattii* have a bipolar mating system with a single *MAT* locus that contains genes encoding homeodomain (HD) transcription factors (*Sxi* genes), pheromones precursors (*MF*) and pheromone receptors (*Ste3*), as well as other genes encoding proteins that may (e.g., *Ste12*, *Ste20*) or may not be related to mating. All other tremellaceous yeasts investigated so far (viz. *Cryptococcus amylolentus*, *C*. *heveanensis*, *Kwoniella magrovensis* and *Tremella mesenterica*) have tetrapolar mating systems with two unlinked loci: one contains two divergently transcribed HD genes (HD locus) and the other (P/R locus) the pheromone and pheromone receptor genes as well as *Ste12*, *Ste20* and other genes found in the *C*. *neoformans MAT* locus [11]. Since the P/R locus is bi-allelic (named A1 and A2) and the HD locus multi-allelic (named B1, B2, etc.), tetrapolar species have multiple mating types. Genes within *MAT* locus display mating type-specific polymorphisms, whereas flanking genes are species specific. Moreover, gene order and orientation within the P/R locus is different in complementary mating types, but synteny is observed in the flanking regions. On the other hand, it has been shown that there is some conservation of gene order in the *MAT* region across species for strains with homologous alleles of the P/R locus [12].

*Cryptococcus flavescens* is a saprobic species that was for many years considered to be a synonym under *C*. *laurentii*. However, analyses of molecular data, namely rDNA gene sequences, indicated that the two species were not conspecific [13]. On the basis of phylogenetic analyses of LSU rRNA gene and ITS sequences, *C*. *flavescens* was found to belong to a clade within the Tremellales that comprised also *C*. *aureus* and *C*. *laurentii*, to which it is physiologically similar [6]. *Cryptococcus flavescens* has been isolated from very distinct locations in Europe, North and South America, and Asia, and from different sources, mainly plant-related substrates and soil, but isolates from animal [14–15] and human-related [6, 16] sources are also known. Some strains of *C*. *flavescens* (cited as *C*. *laurentii*) from wheat and corn were found to conjugate in culture and two groups of opposite mating types were described [17]. However, a complete sexual cycle was never observed. The use of *C*. *flavescens* in biological control has been reported [18] and the genome sequence of a strain (NRRL Y-50378, derived from a strain isolated from wheat anthers in the U.S.A.) capable of suppressing fusarium head blight on wheat was recently released [19]. *Cryptococcus terrestris* was described more recently from the study of strains isolated from soil in the USA and from pigeon droppings and sawdust in Brazil and was found to be the closest phylogenetic relative of *C*. *flavescens* (Crestani et al. 2009). The same authors reported that some strains labelled as *C*. *laurentii* or *C*. *flavescens* are likely to belong to *C*. *terrestris* based on LSU or ITS sequences, but a few others with intermediate phylogenetic positions were not assigned to either *C*. *flavescens* or *C*. *terrestris* (e.g., strains CBS 4926, 8372 and 8645). *Cryptococcus flavescens* and *C*. *terrestris* are hardly distinguishable based on physiology and separation between the two taxa is possible mainly on the basis of LSU and ITS sequence analyses [20]. However, very little is known on the intraspecific genetic variability of either species.

In the present study we examined a total of 59 strains deemed to belong to either *C*. *flavescens* or *C*. *terrestris*, which were obtained from different substrates and geographic locations. We used a multilocus sequencing (MLS) approach to evaluate genetic diversity, reassess species boundaries and obtain clues on their predominant reproduction mode. The following six loci were chosen for sequencing: the D1/D2 domains of the LSU rRNA gene (LSU), the region comprising the ITS spacers and the 5.8S rRNA gene (ITS), the IGS1 spacer flanked by the LSU and 5S rRNA genes, and fragments of three genes encoding the following proteins: the largest subunit of RNA polymerase II (*RPB1*), the translation elongation factor 1 alpha (*TEF1*) and the p21-activated protein kinase (PAK) *STE20*. The MLS approach was complemented by the determination of sequences of putative *MAT* genes from the same strains to assess the potential for sexual reproduction, to provide clues on the mating system of the two species and to gain additional evidence for defining species boundaries. Identification of *MAT* genes was made possible due to the availability of the draft genome sequence of one *C*. *flavescens* strain. The combined MLS and *MAT* gene strategy had been used previously with success to address similar issues in other tremellaceous yeasts of the genus *Kwoniella* by Guerreiro et al. [12]. However, this study is the first to use this approach to identify *MAT* genes for species distantly related to the clades containing *C*. *neoformans* and *Kwoniella* species, and for which only one draft genome is currently available.

## Materials and Methods

### Yeast strains and media

The yeast cultures used in this study are listed in S1 Table. Strains were obtained from the following culture collections: Portuguese Yeast Culture Collection, Portugal (PYCC); Agricultural Research Service Culture Collection, USA (NRRL); Centraalbureau voor Schimmelcultures, The Netherlands (CBS); Culture Collection of Industrial Microorganisms, Slovenia (ZIM); (Agro) Industrial Fungi & Yeasts Collection, Belgium (MUCL); Phaff Yeast Culture Collection, California, USA (UCD). Additional strains were provided by Dr. Deborah Springer (Duke University, USA; isolates collected by Dr. Laura Rusche in Bostwana, DS) and Prof. F.-Y. Bai (State Key Laboratory of Mycology, China; CH). All isolates were grown and maintained on MYP medium at 25°C and 4°C, respectively. MYP medium contained 0.7% (w/v) Malt extract, 0.25% (w/v) Soytone 0.05% (w/v) Yeast Extract and 1.5% Agar.

### Physiological characterization

Physiological tests were performed on sterile 96 well microplates (Nunclon Δ Surface, Denmark) according to Kurtzman et al. [21] and read with StatFax 2100 microplate reader (Awareness Technology Inc., USA) using absorbance measures at 630 nm. Media were prepared according to a protocol available at the CBS website (http://www.cbs.knaw.nl/collections/DefaultInfo.aspx?Page=YeastMethods). The inoculum was grown overnight in liquid MYP medium at 17°C, in Certomat U orbital shaker (Sartorius, Germany) at 48 rpm, and diluted in sterile distilled water for microplate inoculation. Each well contained 200 μl of medium and 5 μl of diluted inoculum (initial absorbance of 0.04). After inoculation, the microplates were sealed with a sealing pellicle (Nunclon Δ Surface, Denmark) and were incubated at 17°C in a Denley Wellwarm 1 (Denley) microplate incubator with shaking, up to three weeks. The ability to grow at 30°C, 35°C and 37°C was determined by inoculating MYP liquid medium with each culture and incubating in water baths at the appropriate temperature for up to five days.

### Mating experiments

To test for sexual compatibility, isolates were grown on MYP agar plates at 17°C for 3 days. Pairs of cell suspensions were then inoculated and mixed together on plates containing CMA, YCB, or MEA medium at 17°C and/or 25°C and examined with a phase-contrast microscope (Zeiss, Germany) for the presence of filaments and sexual structures, up to 12 weeks of incubation. CMA medium contained 1.5% (wt/vol) corn meal agar (Difco) and 0.5% (wt/vol) agar. YCB medium contained 1.17% (wt/vol) yeast carbon base (Difco) and 2% agar. MEA medium contained 2.5% (wt/vol) malt extract (Difco) and 2% (wt/vol) agar.

### Determination of MLS data

Genomic DNA for MLS was obtained using a simplified phenol/chloroform extraction method following cell disruption using glass beads. MLS was based on the following nuclear loci: three regions of the rDNA cistron, namely the D1/D2 domains of the LSU rRNA gene (LSU), the ITS1-5.8S rRNA gene-ITS2 region (ITS), and the intergenic spacer 1 (IGS1); a fragment of the gene encoding the largest subunit of RNA polymerase II (*RPB1*), a fragment of the gene encoding translation elongation factor 1 alpha (*TEF1*) and a fragment of the gene (*STE20*) encoding the homolog of the PAK kinase that functions in the pheromone-activated MAP kinase signaling pathway in *C*. *neoformans* [22]. Four of those loci have been previously used as phylogenetic markers for fungi—e.g., by the Assembling the Fungal Tree of Life (AFTOL) consortium (http://aftol.org)—and IGS1 was used to assess population structure in *C*. *neoformans* and *C*. *gattii* [23]. The *RPB1* and *TEF1* gene fragments contained one and two or three introns, respectively, and the *STE20* fragment contained one intron and a repetitive region. Specific or degenerate primers were ordered or designed as needed, and used to amplify and sequence the six loci. Primers for *STE20* were based on the available draft genome sequence of strain NRRL Y-50378 of *C*. *flavescens* (GenBank accession number CAUG00000000.1) [19]. MLS primer information and PCR conditions are available in the supplemental material (S1 File). Purified PCR amplification products were sequenced by STABVida (Portugal). Primers used for PCR amplification were also used for sequencing, except for ITS and LSU, in which case internal primers ITS1 [24] and NL4 [25], respectively, were used.

### Identification and sequencing of MAT genes

Identification of potential *MAT* genes in *C*. *flavescens* and relatives relied on the design of primers based on the available draft genome sequence of strain NRRL Y-50378 and on primer walking approaches. This strain was deemed to belong to MAT A2 (see Results section). The pheromone cluster (P/R locus) gene organization for the complementary mating-type was predicted based on that of *T*. *mesenterica* DSM 1558 (*MAT* A1). The newly designed primers were tested for PCR amplification and sequencing of putative *MAT* gene fragments with the opposite mating-type strains described by Kurtzman [17], *MAT*α CBS 6473 (= CF59), CBS 6474 (= CF14) and *MAT* **a** CBS 6475 (= CF20) and CBS 6476 (= CF15). Attempts were made to obtain sequences of homologs of several *Cryptococcus MAT* genes comprised in the P/R locus (*STE3*, *STE12*, *CNG04540*, *CNB00600* and *CNB00610*) and in the HD locus (*SXI1* and *SXI2*). When possible, the newly obtained sequences were checked for mating-type-specific polymorphisms, which are expected only for genes within *MAT*. PCR products with the expected size were gel extracted or directly purified using the Illustra GFX PCR DNA and the gel band purification kit (GE Healthcare), according to the manufacturer’s instructions. Primers and PCR amplification conditions used in this study are presented as supplemental material (see S1 File). Primers were synthesized by STABVida (Portugal). Purified PCR amplification products were sequenced by STABVida (Portugal). Primers used for PCR amplification were also used for sequencing, except for *STE3* A1 in which case internal primers STE20_CF_F3 and/or STE3_CF_F3, were used.

### Nucleotide sequence accession numbers

**Fig 1:**
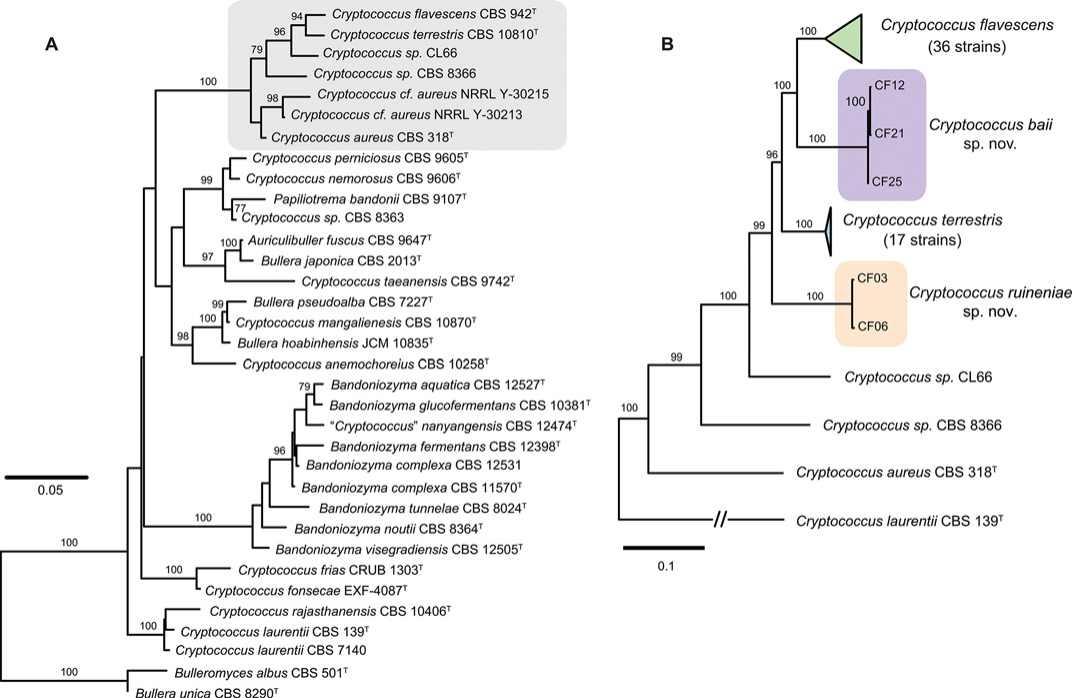
Phylogenetic placement of *Cryptococcus flavescens*, *C*. *terrestris*, and the two novel species *C*. *baii* sp. nov. and *C*. *ruineniae* sp. nov. inferred with maximum likelihood analyses. **(A) phylogenetic position within the *Auricillibuller*-*Papiliotrema* lineage in Tremellales inferred from the analysis of LSU (D1/D2 domains) rDNA and the ITS region; (B) phylogenetic relationships within *C*. *aureus* clade inferred from the analysis of ITS-LSU, IGS1, *RPB1*, and *TEF1* nucleotide sequences.** The numbers given on branches are frequencies (>75%) with which a given branch appeared in 1,000 bootstrap replications. The scale indicates the number of expected substitutions accumulated per site. Accession numbers of nucleotide sequences are provided in S1 and S2 Tables.

Obtained DNA sequences of MLS loci and *MAT* genes were submitted to EMBL under the accession numbers given in S1 Table. Accession numbers of ITS and LSU sequences used in the phylogenetic analysis depicted in Fig. 1A are listed in S2 Table. Sequences of genes CNB00610/CNB00600 (LK024152-LK024159) and CNG04540 (LK024160-LK024170) were also deposited in EMBL.

### Bioinformatic and phylogenetic analyses

Annotation of the different genomic loci was performed with Augustus v2.7 [26] and *Cryptococcus neoformans* prediction model, and checked manually by comparing the obtained predictions with the annotated sequences of *C*. *neoformans* var. *neoformans* JEC21 and/or *C*. *heveanensis* CBS 569. The comparison was done by pair-wise and multiple sequences alignment using MUSCLE software [27] built-in version of MEGA version 5.05 [28] with the default parameters. BLASTn, BLASTp, BLASTx, or tBLASTx (2.2.29 version) searches [29] were also used. Independent alignments and phylogenetic analyses were performed for each locus. Multiple sequence alignments were performed with the genomic sequences using online version of MAFFT algorithm [30] with the default parameters. Alignments of protein-coding gene fragments (*TEF1*, *RPB1* and *STE20*) and the rDNA IGS1 spacer were additionally cured with Gblocks [31] allowing smaller final blocks, gap positions within the final blocks, and less strict flanking positions. The following three datasets were used in phylogenetic analyses: (i) a 5-loci dataset (ITS+LSU and cured IGS1, *TEF1* and *RPB1*) was used to analyse *Cryptococcus aureus* clade with *Cryptococcus laurentii* as outgroup, (ii) a 6-loci dataset (ITS+LSU and cured IGS1, *TEF1*, *RPB1* and *STE20*) was used to analyse the *Cryptococcus flavescens*—*Cryptococcus terrestris* species complex, including the two novel species and (iii) a 6-loci dataset (ITS+LSU, IGS1, *TEF1*, *RPB1* and *STE20*) was used to analyse relationships among *Cryptococcus flavescens* or *Cryptococcus terrestris* strains. Phylogenetic relationships were inferred by the maximum likelihood (ML) method based on the general time reversible (GTR) model with RaxML (version 7.2.8) using raxmlGUI 1.1 [32] and the GTRGAMMA option with 1,000 rounds of bootstrap replicates. To detect recombination in the MLS data set, we applied several recombination tests to evaluate the robustness of our results. To test for evidence of recombination based on phylogenetic compatibilities of nearby polymorphic sites along concatenated sequences, the Φ_*w*_ test [33] implemented in Splitstree 4.10 was used [34]. The test was performed with the default settings of a window size of 100 and k = 2. The partition homogeneity test (PHTest) [35] implemented in PAUP 4.0b10 [36] was used to estimate the degree of incongruence among loci with following parameters: 100 replicates, parsimony as optimality criterion, gaps treated as ‘missing’, tree-bisection-reconnection (TBR) as branch-swapping algorithm and topological constraints not enforced. Additionally, topological similarity between single-gene trees was estimated using *I*_cong_ index, which is based on maximum agreement subtrees, MAST [37]. In order to overcome possible incongruences while combining data from the analysed MLS loci, both in alignments and in trees, phylogenetic network approaches were used to resolve relationships among *Cryptococcus terrestris* and *Cryptococcus flavescens* strains. Therefore, single-gene ML trees were combined into single file and analysed with Splitstree 4.10 [34] using either ConsensusNetwork or SuperNetwork algorithms. SuperNetwork (Z-closure method, mintrees = 4, and 50 iterations) was used to analyse partial trees containing *Cryptococcus terrestris* since two strains CF07 and CF41 lack *STE20* fragment in the alignment. ConsensusNetwork (mean distances and thresholds 0.2 and 0.1) was used to analyse full trees containing only *Cryptococcus flavescens* strains. The index of association (*I*_*A*_) is often applied to measure the degree of linkage disequilibrium among different genotypes, e.g. [38–40]. Standardized *I*_*A*_ and the significance of the null hypothesis of linkage equilibrium (with 10,000 randomizations) were calculated with the program LIAN 3.5 (http://guanine.evolbio.mpg.de/cgi-bin/lian/lian.cgi.pl) using both parametric method and Monte Carlo simulation [41]. Phylogenetic diversity (the sum of the weights for all splits) was estimated using Splitstree 4.10 [34], and the genetic (allelic) diversity was estimated with LIAN 3.5 software. For *MAT* genes analysis, alignments were performed with MEGA 5.05 software [28], using the built-in MUSCLE tool [27] with default parameters. Phylogenetic relationships were inferred using the same methods and parameters described above for MLS analysis.

## Results

### Molecular identification of strains

In the present study a collection of 59 strains originally identified as *Cryptococcus flavescens*, *C*. *terrestris* or *C*. *laurentii* were examined (S1 Table). The latter species was also considered since strains of *C*. *flavescens* can be mistaken for *C*. *laurentii* based on phenotype and *C*. *flavescens* was long considered to be a synonym of *C*. *laurentii* [6]. Selected strains included some of those discussed by Fonseca et al. [6] under *C*. *flavescens*, namely four of the mating strains studied by Kurtzman [17], but also the four strains of *C*. *terrestris* studied by Crestani et al. [20], strains labeled as *C*. *flavescens* or *C*. *terrestris* from different culture collections (ARS, CBS, ZIM, MUCL), isolates from Portugal maintained in the Portuguese Yeast Culture Collection under *C*. *laurentii*, and isolates from recent yeast surveys in China and Botswana identified as *C*. *flavescens* or *C*. *terrestris*. Most strains originated from different environmental sources, mainly plant material, insects and soil, but one clinical isolate previously identified as *C*. *flavescens* (CBS 8645) was also included.

A preliminary analysis based on nucleotide sequences of the two loci most commonly used as barcodes for yeast species identification, i.e., LSU and ITS [42], showed that *C*. *flavescens* and *C*. *terrestris* belong to a strongly supported clade that also included *C*. *aureus* and which is part of a larger lineage within the Tremellales containing taxa such as *Auriculibuller fuscus* and *C*. *laurentii* (Fig. 1A). Two unidentified strains (CL66 and CBS 8366) seem to represent hitherto undescribed taxa in the same clade. However, we have refrained from proposing novel species to accommodate them until additional strains become available for study. Strains showing a close match to either *C*. *flavescens* or *C*. *terrestris*, were studied in more detail (see next sections). Therefore, six DNA loci were amplified and sequenced for these strains (S1 Table). Phylogenetic analysis using concatenated nucleotide sequences of five of those loci showed that out of 57 strains, a total of 35 and 17 strains could be identified as *C*. *flavescens* and *C*. *terrestris*, respectively (Fig. 1B, S1 Table). Five other strains showed only little similarity to either species (Fig. 1B) and seem to represent two novel taxa, for which the names *Cryptococcus baii* sp. nov. (comprising strains CF12, CF21 and CF25) and *Cryptococcus ruineniae* sp. nov. (comprising strains CF03 and CF06) are proposed. Formal descriptions of these species are provided in a separate section.

Phylogenetic analyses of single loci showed different abilities to resolve *C*. *flavescens*, *C*. *terrestris* and the two novel species (S1 and S2 Figs.). LSU was not able to separate the four yeast species clustering them into two large groups (S1 Fig.). ITS showed better discriminating power than LSU but was still insufficient to separate clearly *C*. *flavescens* and one of the novel species (S1 Fig.). Phylogenetic analysis using concatenated sequences of LSU and ITS resulted in reliable topology and resolved the four species (S2A Fig.). However genetic distances were far too small in comparison to the other three gene regions that showed higher variability and discriminating power, in spite of some inconsistencies in topology (S2 Fig.).

### Analyses of MLS data

Phylogenetic analyses of single loci showed that strains of *C*. *flavescens* appear to be more heterogeneous than those of *C*. *terrestris* (Table 1, S3 and S4 Figs.): a total of 63 alleles for all six loci in *C*. *flavescens* compared with 32 in *C*. *terrestris*. However, the genetic (allelic) diversity (H) estimated for the two species was in the same range. Genetic distances in single-gene trees varied substantially, being the lowest in ITS-LSU and the highest in *TEF1* and *STE20* (S3 and S4 Figs.). The position of strains in the obtained trees was not the same among the studied genomic regions. The placement of three *C*. *flavescens* strains (CF01, CF10 and CF46) varied strongly (S3 Fig.). On the other hand, a group of fourteen *C*. *flavescens* strains (CF15, CF16, CF18, CF20, CF22, CF29, CF42, CF43, CF48, CF49, CF54, CF56 and CF57), which includes the type strain (CF22) as well as the WGS strain (NRRL Y-50378), showed high genetic similarity in all studied loci suggesting a possible clonal relationship. Besides this group, a few more pairs of strains displaying identical DNA sequences were observed in this species (S3 Fig.). In contrast, all strains of *C*. *terrestris* were different in the five genomic regions (S4 Fig.). Despite the presence of groups of genetically identical strains, the number of haplotypes observed in *C*. *flavescens* was almost twice as high as that observed in *C*. *terrestris*, 29 and 15, respectively (S3 Table).

**Table 1:** Multi-Locus Sequencing (MLS) results: variability of single gene regions, number of observed alleles, genetic diversity and probability of genetic recombination.

	*C*. *flavescens*	*C*. *terrestris*	*C*. *ruineniae* sp. nov.	*C*. *baii* sp. nov.
Alignments: total length, invariable sites, gaps (bp)				
ITS	435, 429, 1	434, 433, 0	435, 435, 0	435, 434, 0
LSU	554, 541, 0	554, 553, 0	554, 554, 0	554, 554, 0
IGS1	616, 562, 12	657, 653, 0	644, 639, 2	679, 671, 0
*TEF1*	865, 686, 99	800, 679, 7	787, 780, 0	803, 800, 0
*RPB1*	736, 647, 0	734, 689, 0	740, 740, 0	738, 738, 0
*STE20* ^a^	847, 668, 68	847, 771, 64	not sequenced	not sequenced
Number of alleles				
ITS	5	2	1	2
LSU	3	2	1	1
IGS1	10	5	2	3
*TEF1*	16	9	2	3
*RPB1*	14	8	1	1
*STE20* ^a^	15	6	not sequenced	not sequenced
Mean genetic diversity (H) ^b^	0.6124 ± 0.1311	0.6032 ± 0.1376	not applicable	not applicable
Phylogenetic diversity ^d^	0.3064	0.0162	not applicable	not applicable
Φ_*w*_ test (*p-value*) ^c^	Recombination (***)	Recombination (***)	not applicable	not applicable

^a^, estimated for the dataset containing 15 taxa: strains CF07 and CF41 did not yield a PCR product

^b^, mean genetic (allelic) diversity [43] was calculated with LIAN 3.5 [41]

^c^, Φ_*w*_ (PHI, pairwise homoplasy index) test [33] was calculated with SplitsTree 4 software [34];

***, *p* < 0.001

^d^, phylogenetic diversity was calculated with SplitsTree 4 software [34]

**Fig 2:**
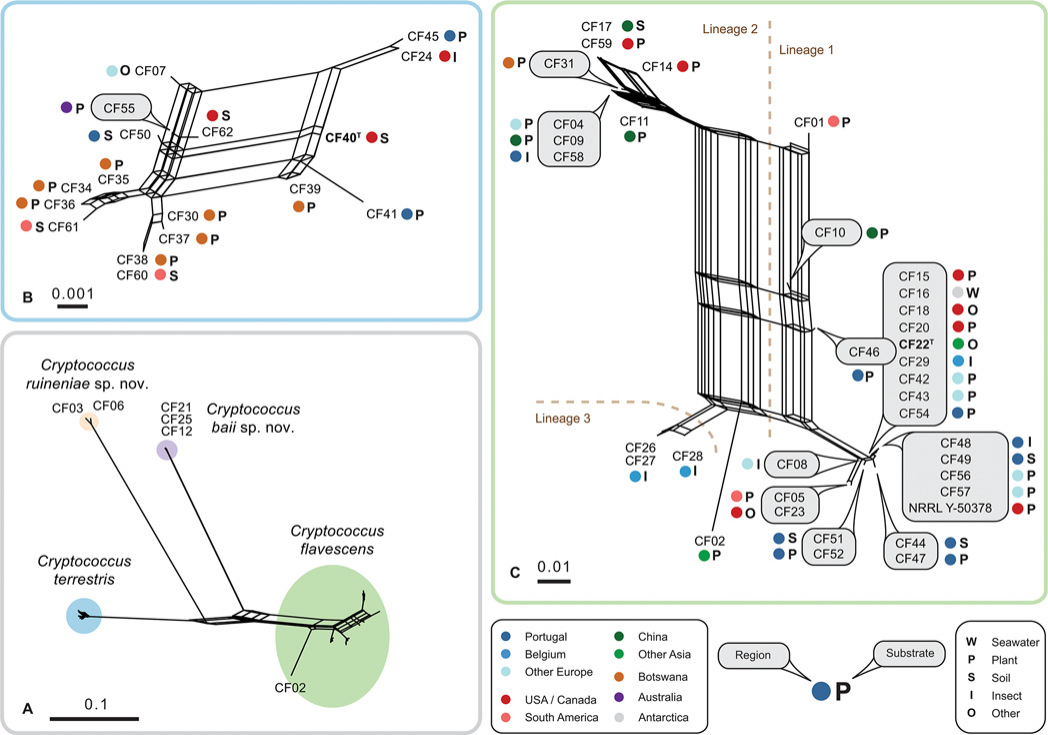
Consensus- and super-networks obtained from the combined analyses of single-gene maximum likelihood trees of ITS-LSU, IGS1, *RPB1*, *TEF1* and *STE20* alignments. **(A) super-network showing relationships between *Cryptococcus flavescens*, *C*. *terrestris*, *C*. *baii* sp. nov. and *C*. *ruineniae* sp. nov.; (B) super-network showing relationships among *C*. *terrestris* strains; (C) consensus network showing relationships among *C*. *flavescens* strains.** The scale indicates mean distance obtained from the analysis of single-gene trees. Type strains are typed in bold. Accession numbers of nucleotide sequences are provided in S1 Table.

Several tests were applied to evaluate the possibility of combining single-gene datasets into a multi-gene phylogenetic analysis. Pairwise comparison of individual alignments with PHTest showed some incongruence suggesting that some loci cannot be properly concatenated for the analysis, namely the protein-coding genes (S4 Table). We also found that out of the three regions comprising the rDNA cistron, ITS and LSU but not IGS1 could be combined for the analyses. Analysis of the topological similarity between single-gene trees using MAST algorithm [37], revealed some incongruence between trees suggesting that topological conflicts between single gene trees cannot be resolved and successfully presented in a single phylogenetic tree but more adequately as a phylogenetic network [44]. Therefore, we attempted to combine single best ML-trees obtained for the five genomic regions (ITS-LSU, IGS1, *RPB1*, *TEF-1* and *STE20*) into three different networks (Fig. 2). In the combined network (Fig. 2A), ML trees were based on alignments containing 989 bp of ITS-LSU, 600 bp of IGS1 (cured with Gblocks, 68% retained), 728 bp of *RPB1* (cured with Gblocks, 93% retained), 806 bp of *TEF-1* (cured with Gblocks, 83% retained), and 775 bp of *STE20* (cured with Gblocks, 91% retained). In the two additional networks that comprised either *C*. *terrestris* (Fig. 2B) or *C*. *flavescens* strains (Fig. 2C), ML trees were calculated using datasets specified in Table 1.

The network analyses revealed a complex structure within *C*. *flavescens* (Fig. 2C), which contrasted with less heterogeneity in *C*. *terrestris* (Fig. 2B) as also suggested by the phylogenetic diversity values estimated for these species (Table 1). Additionally, those analyses confirmed the separation of the two novel species (Fig. 2A). The analysis depicted in Fig. 2C also suggests that, despite its separate position in single gene phylogenetic trees (S3 Fig.), strain CF02 should rather be included in *C*. *flavescens* than considered as a separate species. The distance between CF02 and the nearest group composed of strains CF26, CF27 and CF28 did not exceed the distances between other groups of strains (Fig. 2C). Networks of *C*. *flavescens* and *C*. *terrestris* both display strong reticulations that originate from phylogenetic incongruences between single-gene trees (Fig. 2B and 2C). Based on the relative distance in phylogenetic network analyses and following the approach by Croll and Sanders [39], a total of three genetic lineages were defined in *C*. *flavescens*, whereas strains of *C*. *terrestris* showed a tight reticulation and were thus all assigned to a single lineage (Fig. 2). *C*. *flavescens* lineage 1 had the largest number of strains (24), with the core group consisting of highly similar strains (14 strains, including the type strain), and the three divergent isolates CF01, CF10, CF46 (Fig. 2C), which displayed different placement in single-gene trees (S3 Fig.). Lineage 2 was less heterogeneous and comprised a total of 8 strains. Lineage 3 was represented by three strains exclusively isolated from sawfly larvae in Belgium.

Though some strains clustered close together in the network, no correlation between their origin (location or habitat) and phylogenetic relatedness was observed in *C*. *flavescens* or *C*. *terrestris* (Fig. 2; S1 Table). Even though *C*. *flavescens* lineage 1 was overrepresented by strains isolated in Portugal (eight), these cultures were obtained from remote locations (including the Azores archipelago) and diverse substrates (Fig. 2C). Furthermore, we have found a few examples when isolates originating from the same substrate and location showed distinct haplotypes. Specifically, strains CF26-CF29 (*C*. *flavescens* from sawflies in Belgium) were clustered in lineages 1 and 3 (Fig. 2C), and strains CF34-CF40 (*C*. *terrestris* from Mopane trees in Botswana) were placed in different parts of the network (Fig. 2B).

### Analyses of genetic recombination in *C*. *flavescens* and *C*. *terrestris*

Statistical analyses of single-gene alignments and trees (S4 Table) and strong reticulation in networks (Fig. 2) suggest the presence of genetic recombination in the two species. To detect recombination in the MLS data set, we applied several recombination tests to evaluate the robustness of our results. The Φ_*w*_ test [33] implemented in Splitstree 4.10 [34] suggests recombination being present in both *C*. *flavescens* and *C*. *terrestris* (Table 1). The standardized index of association (*I*_*A*_) and the significance of the null hypothesis of linkage disequilibrium (with 10,000 randomizations) were calculated on the basis of allele assignment presented in S3 Table. Because a population should contain more than three sequences and due to its separate position in phylogenetic analyses, genetic recombination in lineage 3 of *C*. *flavescens* was not determined. Also, the most distant strain of *C*. *flavescens* (CF02) was not assigned to any population. We also applied filtering to our datasets to remove multiple isolates of the same haplotype in both *C*. *flavescens* (29 haplotypes among 36 strains) and *C*. *terrestris* (15 haplotypes among 17 strains). This approach has been applied before to the analyses of genetic linkage disequilibrium in fungi [39–40, 45]. When all isolates of *C*. *flavescens* (either with or without strain CF02) were included in the analyses, both tests showed *I*_*A*_ was significantly different from 0, indicating a high level of linkage disequilibrium and a clonal genetic structure in the global population of *C*. *flavescens* (Table 2). In contrast to *C*. *flavescens*, global population of *C*. *terrestris* is likely to be genetically recombining (Table 2). It has been suggested that the clone-correction (removing replicates of the same sequence type, or haplotype, as repetition of the same sequence type) should be better applied prior to the analysis since the artificially introduced in the dataset clonality, can lead to the detection of linkage disequilibrium and consequently could affect the ability to detect recombination among genotypes, e.g. [39–40, 45]. In our analysis, clone correction did not change the results of the test in the global population of *C*. *flavescens* (Table 2). When clone-corrected single lineages of *C*. *flavescens* were analysed, both tests rejected the null hypothesis of linkage disequilibrium, suggesting the presence of genetic recombination in lineages 1 and 2 but also between strains comprising lineages 1 and 3, and 2 and 3, respectively. No evidence for genetic recombination was observed when strains from lineages 1 and 2 were combined.

**Table 2:** Results of testing for the null hypotheses of linkage disequilibrium for multi-locus allele distribution data for *Cryptococcus flavescens* and *C*. *terrestris*: standardized index of association and the significance values estimated with using both parametric method and Monte Carlo simulation with 10,000 randomizations [41].

Species, lineage	*I*_*A*_ (St)	*p*-values	
		Monte Carlo	Parametric
*C*. *flavescens* (CF), all strains	0.1823	< 0.001	< 0.001
*C*. *flavescens* (CF), filtering applied	0.0945	0.001	< 0.001
*C*. *flavescens* (CF), filtering applied, without CF02	0.0720	0.009	< 0.001
CF lineage 1, all strains	0.1147	0.014	< 0.001
CF lineage 1, filtering applied	0.0049	0.530	1.00
CF lineage 2, all strains	0.0413	0.361	0.441
CF lineages 1 and 2, all strains	0.1600	< 0.001	< 0.001
CF lineages 1 and 2, filtering applied	0.0688	0.018	< 0.001
CF lineages 1 and 3, all strains	0.1448	0.002	< 0.001
CF lineages 1 and 3, filtering applied	0.0267	0.258	0.384
CF lineages 2 and 3, all strains	0.1183	0.058	0.004
*C*. *terrestris* (CT), all strains	0.0670	0.067	0.010

### *MAT* genes identification

**Fig 3:**
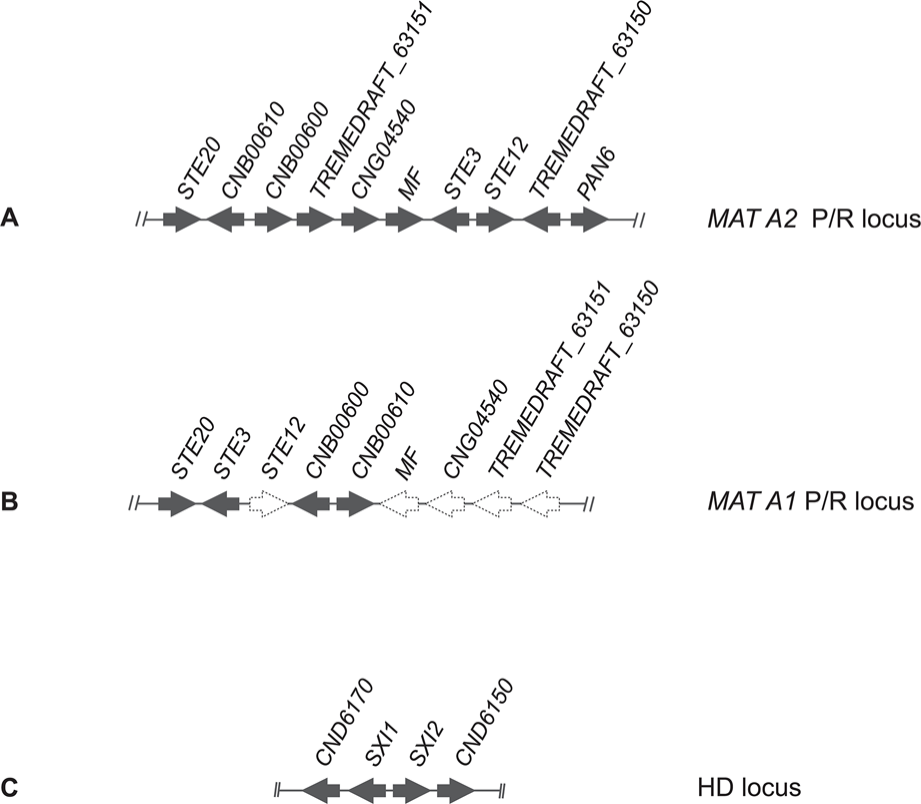
Schematic representation of *Cryptococcus flavescens* gene cluster organization of (A) *MAT* A2 inferred based on the NRRL Y-50378 genome sequence; (B) *MAT* A1 gene organization predicted based on PCR-approaches (dashed arrows); and (C) HD locus inferred based on the NRRL Y-50378 genome sequence.

**Fig 4:**
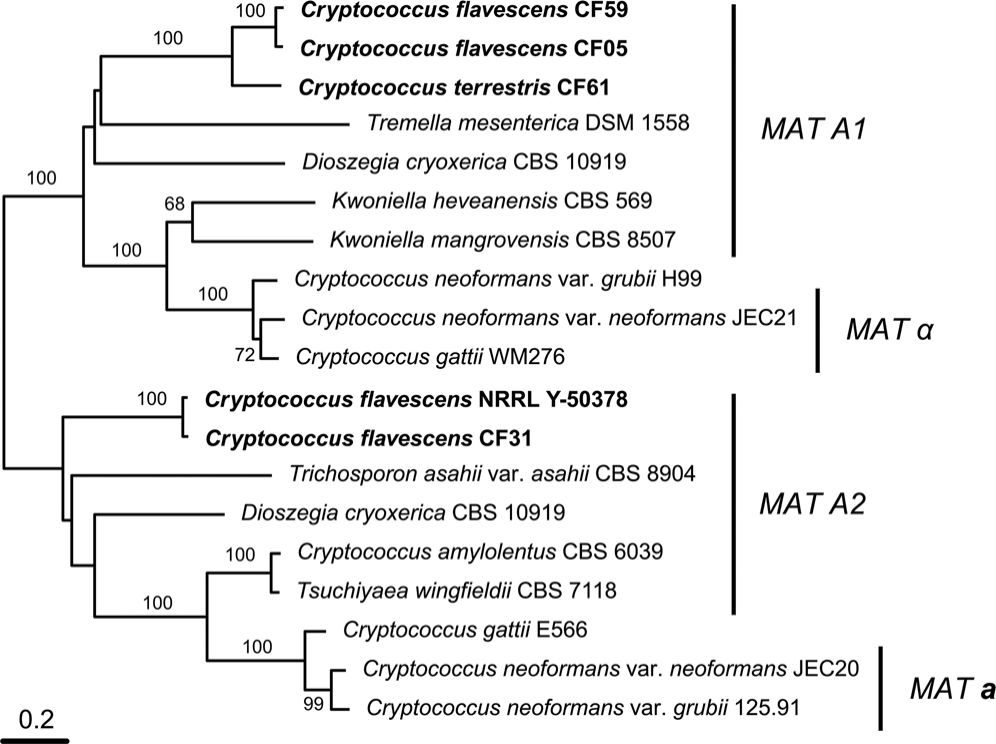
Phylogenetic tree based on complete sequence of *STE3* gene of *Cryptococcus flavescens*, *C*. *terrestris*, and selected members of the Tremellales lineage. The numbers given on branches are frequencies (>70%) with which a given branch appeared in 1,000 bootstrap replications. The scale indicates the number of expected substitutions accumulated per site. Accession numbers of nucleotide sequences are provided in S1 Table.

**Fig 5:**
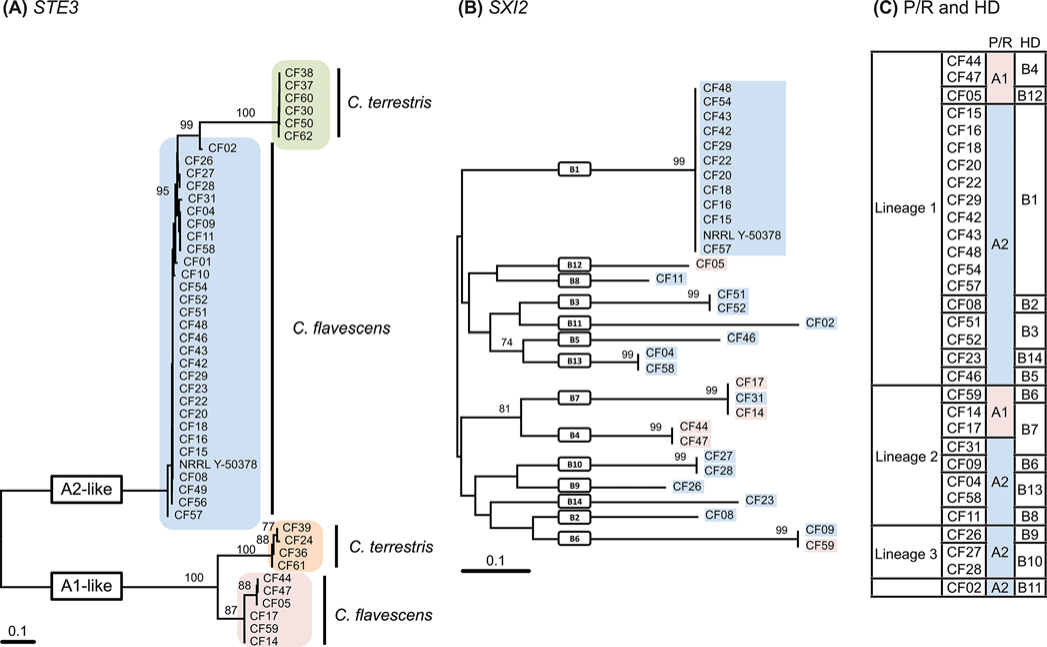
Phylogenetic trees based on (A) partial *STE3* nucleotide sequences of *Cryptococcus flavescens* and *C*. *terrestris*, (B) partial *SXI2* nucleotide sequences of *Cryptococcus flavescens*, and (C) different mating types of *Cryptococcus flavescens* inferred from the analysis of the P/R (A) and HD (B) loci. The numbers given on branches are frequencies (>70%) with which a given branch appeared in 1,000 bootstrap replications. The scale indicates the number of expected substitutions accumulated per site. Accession numbers of nucleotide sequences are provided in S1 Table.

A BLASTx and tBLASTx search [29] with the pheromone receptor (*STE3*) sequences from *Cryptococcus neoformans* JEC20 (AF542530) and JEC21 (AF542531) as queries was used to locate the P/R locus in the NRRL Y-50378 genome sequence. A single contig of ~190 kbp was found (accession number CAUG01000363.1) and sequence annotation enabled us to identify 56 putative genes. Among them we found homologs of genes present within the P/R loci of *K*. *mangrovensis*, *K*. *heveanensis* and *T*. *mesenterica*, namely a mating pheromone precursor gene (*MF*), *STE12*, *STE20*, *CNB00610*, *CNB00600* and *CNG04540*, besides *STE3* (Fig. 3A). Phylogenetic analysis of the latter gene together with homologs from related species revealed that the *STE3* allele of NRRL Y-50378 clustered with the *MAT* A2 alleles, thus suggesting that this strain of *C*. *flavescens* is likely a *MAT* A2 strain (Fig. 4). Since *STE3* and *STE12* are always adjacent and divergently transcribed, we designed a *MAT* A2-specific primer on each gene (see S1 File) based on the NRRL Y-50378 genome sequence to amplify the *STE3* alleles in the other *C*. *flavescens* strains. An amplicon of the expected size (~1860 bp) was obtained for 20 strains belonging to lineage 1, five from lineage 2, the three strains from lineage 3 and the outlier CF02. Partial sequencing of the amplified fragment (~700 bp of *STE3*) confirmed that the *STE3* alleles of these strains are closely related to that of the *MAT* A2 strain NRRL Y-50378 (Fig. 5A). Unsuccessful PCR-amplifications led us to hypothesize that strains CF05, CF44 and CF47 from lineage 1 and CF14, CF17 and CF59 from lineage 2, have *MAT* A1 alleles in their P/R locus. These observations are congruent with the mating-types defined by Kurtzman [17]: CF15 and CF20 (*MAT* **a** strains) were successfully amplified but not CF14 and CF59 (*MAT*α strains).

To check our hypothesis regarding *C*. *flavescens* and to additionally try to determine the P/R locus alleles of *C*. *terrestris*, *C*. *baii* and *C*. *ruineniae* strains, we attempted to amplify and sequence other, more conserved, genes adjacent to *STE3* and *STE12*, namely *STE20*, *CNB00600* and *CNB00610*, with primers based on the NRRL Y-50378 genome sequence. However, when the amplifications were successful, the obtained sequences did not display the mating-type specific polymorphisms expected for genes inside *MAT*. In fact, *STE20* sequences were used for the MLS analysis described in the previous section. We then switched to a different approach based on the observation that gene content and organization of the P/R loci seems to be rather conserved across species outside of the *Cryptococcus* clade among strains belonging to the same mating-type [12]. We thus used a long range PCR-amplification approach to try to amplify *STE3* from the remaining strains, adopting the P/R locus structure of *Tremella mesenterica* DSM 1558 (Fig. 3B) [46] as that of *MAT* A1 strains, whereas that of NRRL Y-50378 was considered as representing the P/R locus structure of *MAT* A2 strains (Fig. 3A). To distinguish between the two possible P/R locus alleles, PCR amplification tests were performed with primers anchored to the *STE20* and *CNB00610* genes (see Text S1). The primer pair STE20_CF_F1 and CNB00610_CF_R1 corresponding to the *MAT* A2 structure in *C*. *flavescens* (Fig. 3A) successfully amplified CF15 and CF20, but failed to amplify CF05, CF14, CF17, CF44, CF47 and CF59, as expected since we hypothesize that the latter strains are *MAT* A1. Moreover, no amplification was observed when the *T*. *mesenterica MAT* A1 structure (Fig. 3B) was tested in *C*. *flavescens MAT* A2 strains (CF15 and CF20) with the pair of primers STE20_CF_F1 and CNB00610_CF_F1. When this primer pair was tested with the six presumed *MAT* A1 strains a fragment of ~9.6 kbp was obtained. The amplicon from strain CF59 was sequenced from the *STE20* end using a primer walking approach, based on primers STE20_CF_F3 and STE3_CF_F3 (see S1 File). As expected, the full *STE3* sequence obtained was more closely related to those of the *MAT* A1 alleles of related species (Fig. 4), thus confirming our hypothesis. *STE3* was amplified (with STE20_CF_F1 and CNB00610_CF_F1) and sequenced (with STE20_CF_F3 and/or STE3_CF_F3) for the other five *MAT* A1 strains and the obtained sequences were similar to that of CF59 (Fig. 5A). Similar approaches were applied to acquire the pheromone receptor sequences from *C*. *terrestris*, *C*. *baii* and *C*. *ruineniae*, but were unsuccessful for the latter two species. However, we were able to amplify and sequence *STE3* from four strains (CF24, CF36, CF39 and CF61) of *C*. *terrestris*, using a primer combination compatible with a *MAT* A1 gene organization (STE20_CF_F1 and CNB00610_CF_F1). Phylogenetic analysis of the obtained sequences confirmed that those four strains had *MAT* A1-like alleles (Fig. 5A). Unsuccessful PCR-amplification with the same primer pair suggested that the P/R locus of the remaining *C*. *terrestris* strains should have *MAT* A2-like alleles. We designed primers in the *CNG04540* (CNG04540_CF_F1 and CNG04540_CF_F2) and *TREMEDRAFT_63150* (TREMEDRAFT_63150_CF_R1) genes, based on the sequence of the homologs of NRRL Y-50378 to acquire the *MAT* A2-like *STE3* allele sequence of *C*. *terrestris*. After several trials with different primers anchored in *STE20*, *CNB00610*, *CNG04540*, *STE3*, *STE12* or *TREMEDRAFT_63150*, we were finally able to acquire the *MAT* A2 *STE3* sequence from six strains of *C*. *terrestris* with the primer combination STE3_CF_F1 and TREMEDRAFT_63150_CF_R1 (S1 Table, Fig. 5A). Results of PCR-amplification tests suggested that the P/R structure in these strains is also concordant with that of the *MAT* A2 allele of *C*. *flavescens* (Fig. 4A). Based on the generated *STE3* sequences, *MAT*-specific primers for each *STE3* allele were designed to determine the mating-type of the remaining *C*. *terrestris* strains. The PCR-amplification results suggest that strains CF34, CF35, CF40 and CF41 are *MAT* A1, and strain CF07 is *MAT* A2 (S1 Table).

A similar approach was used to identify the homeodomain transcription factor genes (HD locus) in the four species. A BLASTx and tBLASTx search [29] with the *SXI1* gene of *Cryptococcus neoformans* JEC21 (AF542531) and *SXI2* of JEC20 (AF542530) as queries was used to locate the HD locus in the NRRL Y-50378 genome sequence. A single contig of ~14.7 kbp was found (accession number CAUG01000094.1). Annotation of the sequence enabled us to identify four genes (Fig. 4C): the two HD genes, *SXI1* and *SXI2*, adjacent and divergently transcribed, and two flanking genes *CND06150* and *CND06170*. Primers were designed in conserved domains of the *SXI1* and *SXI2* sequences of NRRL Y-50378 to amplify a fragment (~1900–2000 bp) containing the 5’ regions of the two genes, as well as the intergenic spacer (see S1 File). PCR amplification tests were successful in most *C*. *flavescens* strains, with the exception of CF01, CF10, CF49 and CF56, but failed in *C*. *terrestris*, *C*. *baii* and *C*. *ruineniae* strains. The amplified fragments were sequenced, and phylogenetic analysis of the partial *SXI2* sequences (~950 bp) revealed a muliallelic HD locus in *C*. *flavescens*, with 14 different alleles identified (Fig. 5B): seven alleles (B1–B5, B12, B14) in lineage 1 strains, four alleles (B6–B8, B13) in lineage 2, two alleles (B9, B10) in lineage 3 and a unique allele (B11) in CF02 (Fig. 5C, S5 Fig.). HD alleles are not shared between lineages and B1 was the most predominant allele identified (11 strains). Separate phylogenetic analyses of the *STE3* and the *SXI2* genes are presented in Fig. 5A and 5B, along with a table combining the allele attributions for the two loci and for each strain (Fig. 5C). Sixteen different mating-types were observed for *C*. *flavescens* alone, and A2B1 was clearly predominant since it was identified in 12 strains of lineage 1, including the type strain and NRRL Y-50378. None of the mating types are shared between lineages and recombination between P/R and HD loci was detected only among lineage 2 strains, namely CF59 (A1B6), CF09 (A2B6), CF14/CF17 (A1B7) and CF31 (A2B7).

### Mating experiments and taxonomic novelties

We performed mating experiments on agar media with several pairs of strains of *C*. *flavescens* and of *C*. *terrestris*, including those that had compatible mating types according to *MAT* gene identification. However, we were unable to observe conjugation between cells or the development of dikaryotic mycelium with clamp connections in any of the crosses. In some cases a few clumps of thin hyphae without clamps but with clusters of blastoconidia were seen. No other structures resembling basidia were detected.

#### *Cryptococcus baii* A. Yurkov, M.A. Guerreiro & Á. Fonseca

Mycobank accession: MB 809088

Etymology: Specific epithet *baii* (N.L. gen. masc. n.), ‘of Bai’, to honour Dr. Fengyan Bai (China) in recognition of his contributions to yeast systematics and ecology, particularly of basidiomycetous yeasts, including the isolation of the type strain of this species.

Standard description: Species belongs to the *C*. *aureus* clade in the Tremellales. Species is apparently anamorphic since no sexual structures have been observed. After 1 week at 25°C colonies on MYP agar are circular, white, ridged, glistening and raised. The texture is butyrous and the margins are entire. After 3 days at 25°C in YM broth the cells are ellipsoid to globose, 4.9–7.1 × 3.7–5.8 μm, and they may be single or with one attached polar bud. Physiological characteristics are listed in S5 Table. Unambiguous identification and phylogenetic placement is based on DNA sequences of the following nuclear loci: ITS (LK023827), IGS1 (LK023890), *RPB1* (LK024012), *TEF1* (LK023951) and *STE20* (LK024031). Strain CH 2.4051 was designated as the type strain and was isolated by F. Bai from an apple tree leaf in the Shandong province, China. This strain has been deposited in the Portuguese Yeast Culture Collection, Portugal as PYCC 6352, and in the Centraalbureau voor Schimmelcultures, The Netherlands, as CBS (*number is pending*). Other strains belonging to this species include: PYCC 5063 from soil in the Azores, Portugal, and UCD 76–106 (PYCC 6524) from a frozen fruit sample in California, USA.

#### *Cryptococcus ruineniae* A. Yurkov, M.A. Guerreiro & Á. Fonseca

Mycobank accession: MB 809089

Etymology: Specific epithet *ruineniae* (N.L. gen. fem. n.), ‘of Ruinen’, to honour Dr. Jakoba Ruinen in recognition of her contributions to yeast ecology, particularly of ‘phyllosphere’ yeasts, including the isolation of the type strain of this species.

Standard description: Species belongs to the *C*. *aureus* clade in the Tremellales. Species is apparently anamorphic since no sexual structures have been observed. After 1 week at 25°C colonies on MYP agar are circular, white, smooth, glistening and raised. The texture is butyrous and the margins are entire. After 3 days at 25°C in YM broth the cells are ovoid, ellipsoid to globose, 4.5–8.0 × 3.5–5.1 μm, and they may be single or with one attached polar bud. Physiological characteristics are listed in S5 Table. Unambiguous identification and phylogenetic placement is based on DNA sequences of the following nuclear loci: ITS (LK023825), IGS1 (LK023888), *RPB1* (LK024010), *TEF1* (LK023949) and *STE20* (LK024023). Strain CBS 4926 was designated as the type strain and was isolated by Jakoba Ruinen from leaves of *Jacquinia aurantiaca* (synonym of *Bonellia macrocarpa*) in Bogor, Indonesia (Ruinen 1963). This strain has also been deposited in the Portuguese Yeast Culture Collection, Portugal as PYCC 6170. One other strain belongs to this species: ML 3703 (CBS 8372; PYCC 6169) collected from water tank of the bromeliad *Quesnelia quesneliana* at Coroa Grande, Brazil.

Although *Cryptococcus* is clearly a polyphyletic genus and the type species is only distantly related to *C*. *flavescens* or *C*. *terrestris*, e.g. [6], we chose to include the novel taxa in this genus until a multilocus study of the members of the *C*. *aureus* and related clades is performed to clarify the generic placement of those species, an endeavor clearly beyond the scope of the present study. The two novel species cannot be distinguished from either *C*. *flavescens* or *C*. *terrestris* based on phenotype (S5 Table). They share with the latter species the ability to grow without added vitamins, an uncommon characteristic among *Cryptococcus* species in the Tremellales [6].

## Discussion

### Species delimitation

Ribosomal DNA-markers, namely LSU and ITS, have successfully replaced physiological tests for yeast species identification, e.g. [42, 47–48]. Specifically, it has been suggested that yeasts showing more than 1% divergence in any of two regions are likely to represent different species. This conclusion was inferred from the available data and was originally considered as a recommendation rather than a rule. Nevertheless, it has been converted into a principle widely accepted by the yeast community (sometimes referred to as “1% rule”) and LSU and ITS served as the first DNA-barcodes applied to this large group of Fungi. In the last two decades, different DNA loci such as ribosomal SSU, *RPB1*, *TEF-1*, *β-Tub*, *ACT*, *CAM* and *MCM7* genes have been applied to species identification in different fungal clades, e.g. [49–54]. In a recent study Schoch et al. [55] demonstrated that ITS was generally superior to LSU as the universal DNA-barcode for Fungi for having the highest probability of successful identification across the fungal kingdom, together with the most clearly defined barcode gap between inter- and intraspecific variation. Among the analysed protein-coding genes *RPB1* showed least problems in amplification and sequencing, and provided high levels of species discrimination [55].

In the present study, we showed the utility of an MLS approach that combined rDNA cistron regions with protein-coding genes and involved an enlarged strain set for successful yeast species delimitation within the Tremellales (Fig. 2), namely enabling the clear separation between *C*. *flavescens* and *C*. *terrestris*. Furthermore, we demonstrate that the *C*. *flavescens*—*C*. *terrestris* complex contains a total of four cryptic species, including the two novel species described herein. These species display similar physiological properties and are hardly distinguishable based solely on LSU and ITS sequences but rather on the multi-locus dataset. Besides ITS and LSU, IGS1 and *RPB1* showed least problems in amplification and sequencing in the present study. Sequencing of *MAT* genes, namely *STE3*, corroborated the separation of *C*. *flavescens* and *C*. *terrestris* at the species level and confirmed the conspecificity of the *C*. *flavescens* strains in spite of the genetic heterogeneity observed in some of the genes used in the MLS analysis.

Phylogenetic analyses of single DNA-loci revealed the problem common to other MLS studies that sequences of single loci cannot always be concatenated as suggested by PHTest. Disagreement between several DNA loci and its influence on phylogenetic relationships among yeast has been shown for Saccharomycetales [56]. The latter authors suggested using phylogenetic networks rather than phylogenetic trees to explore contradictory phylogenetic relationships among different yeast genera. In the present study, we show the usefulness of phylogenetic networks to resolve a cryptic species complex. Lachance et al. [57–58] applied parsimony network analyses to rDNA ITS-LSU datasets to delimit closely related species of ascomycetous yeasts. However, in our study this gene region was not variable enough. Because the variability level differed substantially between different loci, we applied a consensus network approach to combine results from single gene trees (Fig. 2). With this analysis we show that intraspecific variability can vary substantially between closely related species as exemplified with *C*. *flavescens* (genetically more heterogenous) and *C*. *terrestris* (Table 1, Fig. 2). Therefore, the single cut-off rule such as commonly applied 90% to 99% sequence similarity values of ITS-based molecular OTUs, e.g. [59–61], can hardly be applied to large yeast lineages as well as to single clades.

Even though data regarding strains of *C*. *flavescens* deposited in public collections suggested this species to be frequent in many different natural substrates, our study indicates that the majority of isolates originated from plant-related habitats such as phylloplane and plant-grazing insects (S1 Table). *C*. *terrestris* was known from soil, pigeon droppings and sawdust [20], but in the present study we have identified a substantial number of isolates associated with plant material (S1 Table). The single clinical isolate included in this study (CF07) was previously identified as *C*. *flavescens* [13], but was found to belong to *C*. *terrestris*. Moreover, we resequenced the LSU and ITS loci and found that the sequences of *C*. *terrestris* strains (including the type culture) determined by Crestani et al. [20] and deposited in Genbank (EU200782 = NR_073350) were actually incorrect. Analysis of strain origin of *C*. *flavescens* and *C*. *terrestris* showed they are geographically widespread (Fig. 2; S1 Table). Moreover, we could not find hardly any correlation between genotype and geography (except for lineage 3 of *C*. *flavescens*) and the two species appear to have global populations with little, if any endemism, a situation observed in human pathogenic fungi such as *C*. *neoformans* / *C*. *gattii* [5] and *Aspergillus fumigatus* [45]. Because distribution of both species included plant-related substrates that were in some cases isolated from the same locations (viz., strain CF31 and strains CF34-39 from Mopane trees in Botswana; strains CF50 and CF51 from soil in Portugal; strains CF42 and CF43 from grapes in Slovenia), future studies should address the question of whether or not these two sister species co-occur in nature. The almost identical physiological profiles of *C*. *flavescens* and *C*. *terrestris* are in agreement with occupation of similar niches by the two species. The two novel species, *C*. *baii* and *C*. *ruineniae*, are represented by very few strains that were isolated mainly from plant-related substrates albeit from very distant geographical locations (S1 Table). *C*. *ruineniae* may be restricted to regions with tropical climate since the two strains were obtained from tropical plants.

### Asexual versus sexual species

Several molecular studies have shown that yeasts of the genus *Cryptococcus* are phylogenetically intermingled with members of sexual taxa within the Tremellales, such as *Auriculibuller*, *Cuniculitrema*, *Fibulobasidium*, *Papiliotrema*, and *Tremella*, e.g. [6, 9, 42]. Moreover, several species of *Cryptococcus* were shown to be able to reproduce sexually upon crossing of compatible strains on culture media, viz. *C*. *amylolentus* [62], *C*. *heveanensis* [46], *K*. *mangrovensis* [63] and most notably *C*. *neoformans* [64]. However, the direct observation of the sexual phase in culture may require conditions that cannot be reproduced in vitro, as in the case of members of the genera *Bulleribasidium*, *Cuniculitrema*, *Sirobasidium*, *Rhynchogastrema* and *Tremella* that are parasitic on other fungi and lichens [9, 65–67]. Molecular techniques, in particular multi-locus sequence (MLS) analyses (sometimes also referred to as multi-locus sequence typing, MLST), may circumvent those limitations and reveal the presence of genetic recombination among fungal populations [39–40, 68–69]. Specifically, MLSA / MLST approaches helped to show recent recombination events within the human pathogen *C*. *gattii* [10, 70]. In the present study we have adapted MLSA approach for studying genetic recombination within non-pathogenic yeast species. Therefore, we have analysed sequences from five genomic regions for 52 strains belonging to the two yeast species *Cryptococcus flavescens* and *C*. *terrestris*. Incongruences between single-gene trees and the presence of reticulation in the phylogenetic network analyses depicted in Fig. 2 already suggested the existence of genetic exchange within *C*. *flavescens* and *C*. *terrestris*. In order to confirm the presence of genetic recombination we assessed linkage disequilibrium (standardized *I*_*A*_ index) and genetic recombination (Φ_*w*_ test) for a total of 63 and 32 alleles, respectively derived from the analysis of the five loci. While results of the Φ_*w*_ test suggested genetic recombination being present in both species, testing for linkage equilibrium did not yield a clear result. After removing genetically identical haplotypes (clone correction) [39, 40, 45], we observed statistical support for recombination (i.e. existence of linkage equilibrium) among all *C*. *terrestris* but not among *C*. *flavescens* strains. Within *C*. *flavescens*, linkage equilibrium was not observed in the global population but within genetic lineages 1 and 2, as suggested by low *I*_*A*_ and high *p-*values (Table 2). The presence of genetic recombination was also suggested in combination of strains of lineage 3 with those of lineages 1, while the population structure within of strains of lineages 2 and 3 was close to clonal. No genetic recombination of strains of the two largest lineages 1 and 2 was observed in the analysis of linkage equilibrium (Table 2). On the other we found what appears to be a larger clonal group within lineage 1 of *C*. *flavescens*, which comprises isolates from very different geographic origins (Fig. 2). Although not fully conclusive, our results suggest that *C*. *terrestris* consists of a single recombining population and that *C*. *flavescens* consists of different genetic lineages, which show different levels of recombination but show also clonal expansion, at least within lineage 1.

### *MAT* loci and mating system

The potential for recombination within *C*. *flavescens* and *C*. *terrestris* was further demonstrated by the identification of complementary alleles of the pheromone receptor genes (*STE3*) in both species. In fact, primers designed based on the NRRL Y-50378 genome sequence allowed us to sequence the *STE3* gene from *C*. *flavescens MAT* A2 strains, and long range PCR amplification approaches allowed us to amplify and sequence the *STE3* gene from the *C*. *flavescens MAT* A1 strains and *C*. *terrestris MAT* A1 and *MAT* A2 strains (S1 Table). Phylogenetic analyses (Figs. 4 and 5) suggest the presence of biallelic P/R loci in both *C*. *flavescens* and *C*. *terrestris*, with a predominance of *MAT* A2 strains in *C*. *flavescens* (30 out of 36 strains) and an even distribution in *C*. *terrestris*. We also demonstrated the presence of a multi-allelic HD locus in *C*. *flavescens* and were able to identify 16 different mating types (Fig. 5). Although none of the mating types were shared by strains of different lineages, we found evidence for recombination between the P/R and HD loci within lineage 2, which correlates with the support for linkage equilibrium as measured by the index of association (Table 2). No correlation was found between mating-type and geographic origin of the strains. For example, A2B1 mating-type strains were isolated in Europe (Belgium, Germany, Portugal and Slovenia), USA, Antarctica and Japan; and among strains isolated in Portugal, five different mating-types (A1B4, A2B1, A2B3, A2B5 and A2B13) were identified. Although we demonstrated a biallelic P/R locus in *C*. *terrestris*, further analysis of the HD locus still needs to be performed to determine the mating system in this species. *C*. *baii* and *C*. *ruineniae* mating systems remain to be unveiled, since we were unable to obtain *MAT* gene sequences from both loci in strains of those species.

Our results for *C*. *flavescens* provide support to the findings of Kurtzman [17] who reported mating experiments with some of the strains used in the present study that resulted in the formation of true hyphae devoid of clamp connection but with chlamydospore-like structures. Original illustrations displayed germination of chlamydospores (interpreted as possible teliospores) and led Kurtzman [17] to suggest that *C*. *flavescens* (cited as *C*. *laurentii*) was a sexual species. Moreover, our results demonstrate that two pairs of compatible strains studied by Kurtzman [17] have complementary mating types: *MAT* **a** strains CF15 and CF20 were found to be A2B1, and *MAT*α strains CF14 and CF59 were found to be A1B7 and A1B6, respectively. However, our attempts at crossing strains from the same lineage and displaying compatible mating type types on culture media were unsuccessful. Those negative results may be actually due to the need of specific media components or conditions that we were unable to provide and not necessarily to the intrinsic inability of those strains to mate.

Annotation of the *C*. *flavescens* genome and sequencing of the *STE3* and *SXI* genes further demonstrated the existence of a tetrapolar mating system in this species, with separate P/R and HD loci. This finding provides further support to the hypothesis that the tetrapolar system is prevalent in the Tremellales and that the bipolar system is so far restricted to the taxa in the *C*. *neoformans* species complex [11–12]. Our results further suggest that gene organization in the P/R genomic region is essentially maintained in both *C*. *flavescens* and *C*. *terrestris* mating-types, and in *T*. *mesenterica MAT* A1. This finding concurs with the recent results of Guerreiro et al. [12] regarding remarkable synteny of *MAT* P/R region of species in the *Kwoniella* clade with *C*. *heveanensis* and *T*. *mesenterica*. This conservation of synteny in species that are phylogenetically distant may help in the identification of *MAT* loci in other species in the Tremellales.

## Conclusions

In summary, our findings provide strong evidence to suggest that two environmental non-pathogenic *Cryptococcus* species thought to be asexual, *C*. *flavescens* and *C*. *terrestris*, are most likely recombining in nature as demonstrated by the analyses of MLS data as well as from the identification of *MAT* genes in both species, in spite of our inability to demonstrate direct mating in culture.

In this study we present modified approach towards resolving intraspecific structure and genetic recombination in environmental yeast populations. Our results show the usefulness of phylogenetic network analyses over conventional single-gene phylogenetic trees, as the latter cannot be utilized for analyses of incongruent datasets. Our study show superior performance of multi-gene phylogenetic analyses over commonly used rDNA ITS-LSU fragment but also cautions of using pre-defined cut-offs for species delimitation in Tremellomycetes since no fixed criterion based on sequence variability and species (OTUs) delimitation can be inferred even for closely related species as exemplified with *C*. *flavescens* and *C*. *terrestris*.

## Supporting Information

S1 Fig Maximum likelihood trees of (A) LSU and (B) ITS alignments showing phylogenetic relationships among members of the *Cryptococcus aureus* clade. The tree is rooted with *C*. *laurentii* CBS 139^T^. The numbers given on branches are frequencies (>75%) with which a given branch appeared in 1,000 bootstrap replications. The scale indicates the number of expected substitutions accumulated per site. Accession numbers of nucleotide sequences are provided in S1 and S2 Tables.
(EPS)

S2 Fig Phylogenetic placement of *Cryptococcus flavescens*, *C*. *terrestris*, *C*. *baii* sp. nov. and *C*. *ruineniae* sp. nov. inferred with maximum likelihood analyses of (A) ITS-LSU, (B) IGS1, (C) *RPB1*, and (D) *TEF1* alignments. Other details as for S1 Fig.
(EPS)

S3 Fig Maximum likelihood trees of (A) ITS-LSU, (B) IGS1, (C) *RPB1*, (D) *TEF1* and (E) *STE20* alignments showing phylogenetic relationships among strains of *Cryptococcus flavescens*. Genetic lineages are given according to Fig. 2. Other details as for S1 Fig.
(EPS)

S4 Fig Maximum likelihood trees of (A) ITS-LSU, (B) IGS1, (C) *RPB1*, (D) *TEF1* and (E) *STE20* alignments showing phylogenetic relationships among strains of *Cryptococcus terrestris*. Other details as for S1 Fig.
(EPS)

S5 Fig Consensus networks (mean distances, threshold 0.2) obtained from the combined analyses of single-gene maximum likelihood trees of ITS-LSU, IGS1, *RPB1*, *TEF1* and *STE20* alignments for *Cryptococcus flavescens* strains. The scale indicates mean distance obtained from the analysis of single-gene trees. Genetic lineages (L1, L2 and L3) are given according to Fig. 2. Clone-corrected results of genetic recombination analyses (standardized *I*_*A*_ index and *p*-values) are given according to Table 2. Different mating types of *C*. *flavescens* strains are given according to Fig. 5.
(EPS)

S1 File Additional details to methods for amplification and sequencing of *MAT* genes. (DOCX)

S1 Table List of yeast strains used in this study and accessions numbers of generated gene sequences. (XLS)

S2 Table Accession numbers of LSU and ITS nucleotide sequences used in the phylogenetic analyses, Fig. 1A. (XLS)

S3 Table Gene alleles coding used for genetic recombination analyses (Table 2) within *Cryptococcus flavescens* and *C*. *terrestris* strains. (XLSX)

S4 Table Results of partition homogeneity test (PHTest) and topological congruence test (MAST algorithm) for single-gene alignments and maximum likelihood trees. (XLSX)

S5 Table Physiological characteristics of *Cryptococcus flavescens*, *C*. *terrestris*, *C*. *baii* sp. nov. and *C*. *ruineniae* sp. nov. (XLSX)
