# Correction: Multigene Assessment of the Species Boundaries and Sexual Status of the Basidiomycetous Yeasts *Cryptococcus flavescens* and *C*. *terrestris* (Tremellales)

**DOI:** 10.1371/journal.pone.0126996

**Published:** 2015-04-24

**Authors:** Andrey Yurkov, Marco A. Guerreiro, Lav Sharma, Cláudia Carvalho, Álvaro Fonseca

In the Funding section, a second grant number from the funder FCT—Fundação para a Ciência e a Tecnologia, Portugal is missing. The second grant number is: PTDC/BIA-BIC/4585/2012.

The complete, correct funding information is as follows: This work was supported by funds from FCT—Fundação para a Ciência e a Tecnologia, Portugal (www.fct.pt), project PTDC/BIA-MIC/113051/2009 and PTDC/BIA-BIC/4585/2012. The funders had no role in study design, data collection and analysis, decision to publish, or preparation of the manuscript.
