# Supplementary material for: Multigene Assessment of the Species Boundaries and Sexual Status of the Basidiomycetous Yeasts Cryptococcus flavescens and C. terrestris (Tremellales)
Source: PLoS One. 2015 Mar 26;10(3):e0120400. doi: 10.1371/journal.pone.0120400 (PMC4374795; doi:10.1371/journal.pone.0120400)

**Multigene assessment of the species boundaries and sexual status of the basidiomycetous yeasts *Cryptococcus flavescens* and *C. terrestris* (Tremellales)**

Andrey Yurkov, Marco A. Guerreiro, Lav Sharma, Cláudia Carvalho, Álvaro Fonseca

Supporting Information Text S1

PCR amplification and sequencing

The reaction mixture used for each standard PCR amplification contained 1x DreamTaq™ Buffer (Fermentas), 0.2 mM dNTPs (Fermentas), 0.4 μM of each primer, 0.04 U/μl of DreamTaq™ DNA Polymerase (Fermentas) and 8 ng of DNA in a total volume of 50 μl. The reaction mixture used for Long Range PCR amplification contained 1x High Fidelity PCR Buffer (Invitrogen), 2 mM MgSO4 (Invitrogen), 0.2 mM dNTPs (Fermentas), 0.4 μM of each primer, 1U of Platinum® Taq DNA Polymerase High Fidelity (Invitrogen). The complete list of primers designed in this study is provided in Table A, and their location is represented in Figures A and B.

MLS loci amplification

The ITS-D1/D2 rDNA region was amplified with primer pair ITS5 (White et al., 1990) and LR6 (Vilgalys and Hester 1990). The PCR amplification consisted of an initial denaturation for 5 min at 95 ºC, followed by 35 cycles of 30 sec denaturation at 95 ºC, annealing for 30 s at 50 ºC and extension for 2 min at 72 ºC. The final extension step was carried out at 72 ºC for 7 min.

The *IGS1* region was amplified with primer pair LR12R (designed by AFTOL consortium, James et al. 2006) and 5SR2 (5’- CTCCBTGGTACTAACYGAGCG -3’), designed based on *Cryptococcus neoformans* JEC21 (AE017342) and H99 (CP003821), *Cryptococcus gattii* (CP000287) and *Tremella mesenterica* DSM 1558 (JGI database) 5S gene sequences. The PCR amplification consisted of an initial denaturation for 4 min at 96 ºC, followed by 10 cycles of 30 sec denaturation at 96 ºC, annealing for 30 sec at 58 ºC and decreasing 0.5 ºC per cycle, extension for 1 min at 72 ºC, followed by 30 cycles of 30 sec denaturation at 96 ºC, annealing for 1 min at 54 ºC and extension for 1 min at 72 ºC. The final extension step was carried out at 72 ºC for 7 min.

*RPB1* was amplified with primer pair RPB1_Af (Stiller and Hall 1997) and RPB1_Cr (Matheny *et al*. 2002). The PCR amplification consisted of an initial denaturation for 2 min at 96ºC, followed by 35 cycles of 20 sec denaturation at 96ºC, annealing for 40 s at 52ºC and extension for 1 min at 72ºC. The final extension step was carried out at 72ºC for 7 min.

*TEF1* was amplified with primer pair EF1α_F1 and EF1α_R1 (Guerreiro *et al.* 2013). The PCR amplification consisted of an initial denaturation for 4 min at 95 ºC, followed by 20 cycles of 1 min denaturation at 95 ºC, annealing for 1 min at 65 ºC and decreasing 0.5 ºC per cycle, extension for 1 min at 72 ºC, followed by 35 cycles of 1 min denaturation at 95 ºC, annealing for 1 min at 55 ºC and extension for 1 min at 72 ºC. The final extension step was carried out at 72 ºC for 10 min.

*STE20* was amplified with primer pair STE20_CF_F1 (5’- CCA ACT TTG TGC ATC AAG TAC ACG -3’) and STE20_CF_R1 (5’- CTC CAT GTA CTC CAT GAC GAC CC -3’), designed based on NRRL Y-50378 genome sequence. The PCR conditions were the same as for the ITS-D1/D2 rDNA region.

MAT genes amplification

*SXI1*-*SXI2* regions (Figure A) were amplified with the primer pairs SXI1_CF_F1 (5’- CGT GTC TTT AAT GTC GGT CCA CC -3’) or SXI1_CF_F2 (5’- CCA TGA CCC AAC CCC ATA CTC C -3’) and SXI2_CF_R1 (5’- CCC TCT GTT CCG CTT GTT CTG -3’), designed based on the NRRL Y-50378 genome sequence. For the SXI1_CF_F1 and SXI2_CF_R1 primer pair, the PCR amplification consisted of an initial denaturation for 5 min at 95 ºC, followed by 9 cycles of 30 sec denaturation at 95 ºC, annealing for 30 sec at 60 ºC and decreasing 1 ºC per cycle, extension for 2 min at 72 ºC, followed by 35 cycles of 30 sec denaturation at 95 ºC, annealing for 30 sec at 50 ºC and extension for 2 min at 72 ºC. The final extension step was carried out at 72 ºC for 7 min. For the SXI1_CF_F2 and SXI2_CF_R1 primer pair, the PCR amplification consisted of an initial denaturation for 5 min at 95 ºC, followed by 35 cycles of 30 sec denaturation at 95 ºC, annealing for 30 s at 50 ºC and extension for 2 min 20 sec at 72 ºC. The final extension step was carried out at 72 ºC for 7 min.

The CNB00610 and CNB00600 regions (Figure B) were amplified with the primer pair CNB00610_CF_F1 (5’- GGC TGA GAA GAG TAG ATT TTG GCG -3’) and CNB00600_CF_R1 (5’- CGC CAA AAT CTA CTC TTC TCA GCC -3’), designed based on the NRRL Y-50378 genome sequence. The PCR conditions were the same as for the ITS-D1/D2 rDNA region.

In *C. flavescens* and *C. terrestris*, *STE3* A1 allele was amplified with primer pair STE20_CF_F1 (5’- GCG ATC AAG CGA AGA TAT CG -3’) and CNB00610_CF_F1 (5’- GGC TGA GAA GAG TAG ATT TTG GCG -3’), designed based on NRRL Y-50378 genome, and sequenced with STE20_CF_F3 (5’- CTG ATG AAC AGC ACG AGT TCC -3’), and/or STE3_CF_F3 (5’- CTG GCG ATC AGR ACG GCG AG -3’), designed based on primer walking of the strains CF59 and CF61 (Figure B). *C. flavescens* *STE3* A2 allele was amplified and sequenced with primer pair STE3_CF_F1 (5’- GCG ATC AAG CGA AGA TAT CG -3’) and STE12_CF_R1 (5’- CTC GAA GCG GAA TGA GAG G -3’), designed based on the NRRL Y-50378 genome. *C. terrestris*, *STE3* A2 allele was amplified with primer pair STE3_CF_F1 and TREMEDRAFT_63150_CF_R1 (5’- GGA CGA AGG ATA ACT GTT CAG TGG -3’), designed based on NRRL Y-50378 genome sequence and sequenced with STE3_CF_F1. A Long Range PCR approach was used with the primer pair STE20_CF_F1 and CNB00610_CF_F1, which consisted of an initial denaturation for 30 min at 94 ºC, followed by 35 cycles of 30 sec denaturation at 94 ºC, annealing for 30 s at 50 ºC and extension for 12 min at 68 ºC. The final extension step was carried out at 68 ºC for 30 min. For the primer pair STE3_CF_F1 and STE12_CF_R1, the PCR amplification consisted of an initial denaturation for 5 min at 95 ºC, followed by 35 cycles of 30 sec denaturation at 95 ºC, annealing for 30 s at 52 ºC and extension for 2 min at 72 ºC. The final extension step was carried out at 72 ºC for 7 min. A Long Range PCR approach was used with the primer pair STE3_CF_F1 and TREMEDRAFT_63150_CF_R1, which consisted of an initial denaturation for 30 min at 94 ºC, followed by 35 cycles of 30 sec denaturation at 94 ºC, annealing for 30 s at 50 ºC and extension for 8 min at 68 ºC. The final extension step was carried out at 68 ºC for 30 min.

References

Guerreiro MA, Springer DJ, Rodrigues JA, Rusche LN, Findley K, et al. (2013) Molecular and genetic evidence for a tetrapolar mating system in the basidiomycetous yeast *Kwoniella mangrovensis* and two novel sibling species. *Eukaryotic Cell* 12: 746-760.

James TY, Kauff F, Schoch CL, Matheny PB, Hofstetter V, et al. 2006. Reconstructing the early evolution of the fungi using a six gene phylogeny. *Nature* 443: 818-822.

Matheny PB, Liu YJ, Ammirati JF, Hall BD. 2002. Using *RPB1* sequences to improve phylogenetic inference among mushrooms (Inocybe, Agaricales). *American Journal of Botany* 89: 688-698.

Stiller JW, Hall BD. 1997. The origin of red algae: Implications for plastid evolution. *Proceedings of the National Academy of Sciences of the United States of America* 94: 4520-4525.

Vilgalys R, Hester M. 1990. Rapid genetic identification and mapping of enzymatically amplified ribosomal DNA from several *Cryptococcus* species. *Journal of Bacteriology* 172: 4238-4246.

White TJ, Bruns T, Lee S, Taylor JW. 1990. Amplification and direct sequencing of fungal ribosomal RNA genes for phylogenetics. In: Innis MA, Gelfand DH, Sninsky JJ, White TJ (eds.) PCR Protocols: A Guide to Methods and Applications, pp. 315-322, Academic Press, Inc., New York.

**Table A. List of the primers designed in this study.**


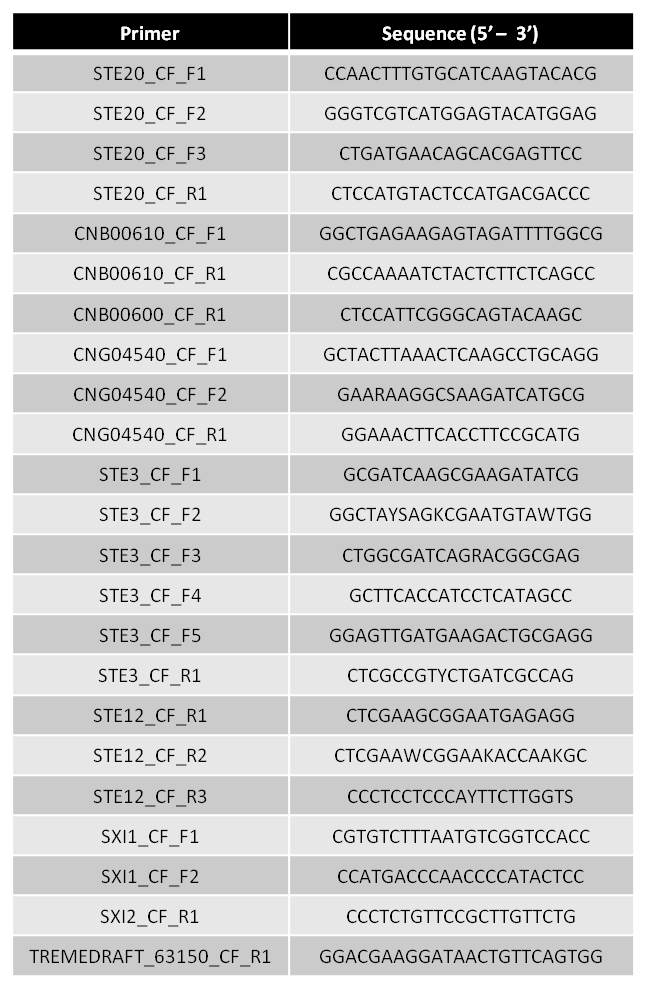


**Fig. A. Primers location in *C. flavescens* HD locus.**


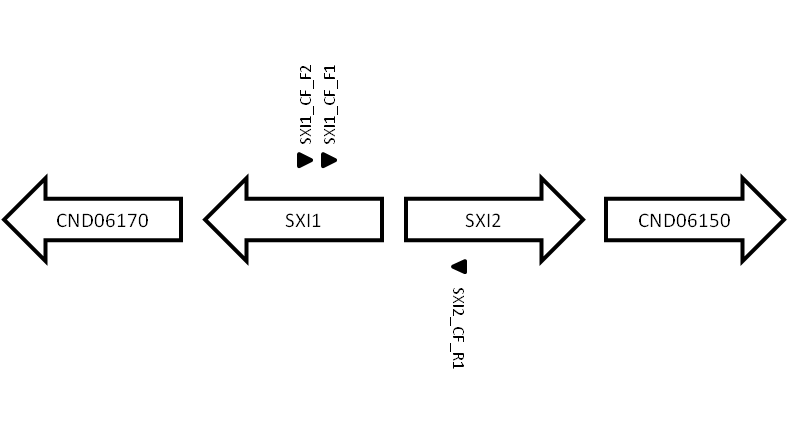


**Fig. B. Primers location in *C. flavescens* P/R loci (*MAT* A2 and *MAT* A1)**


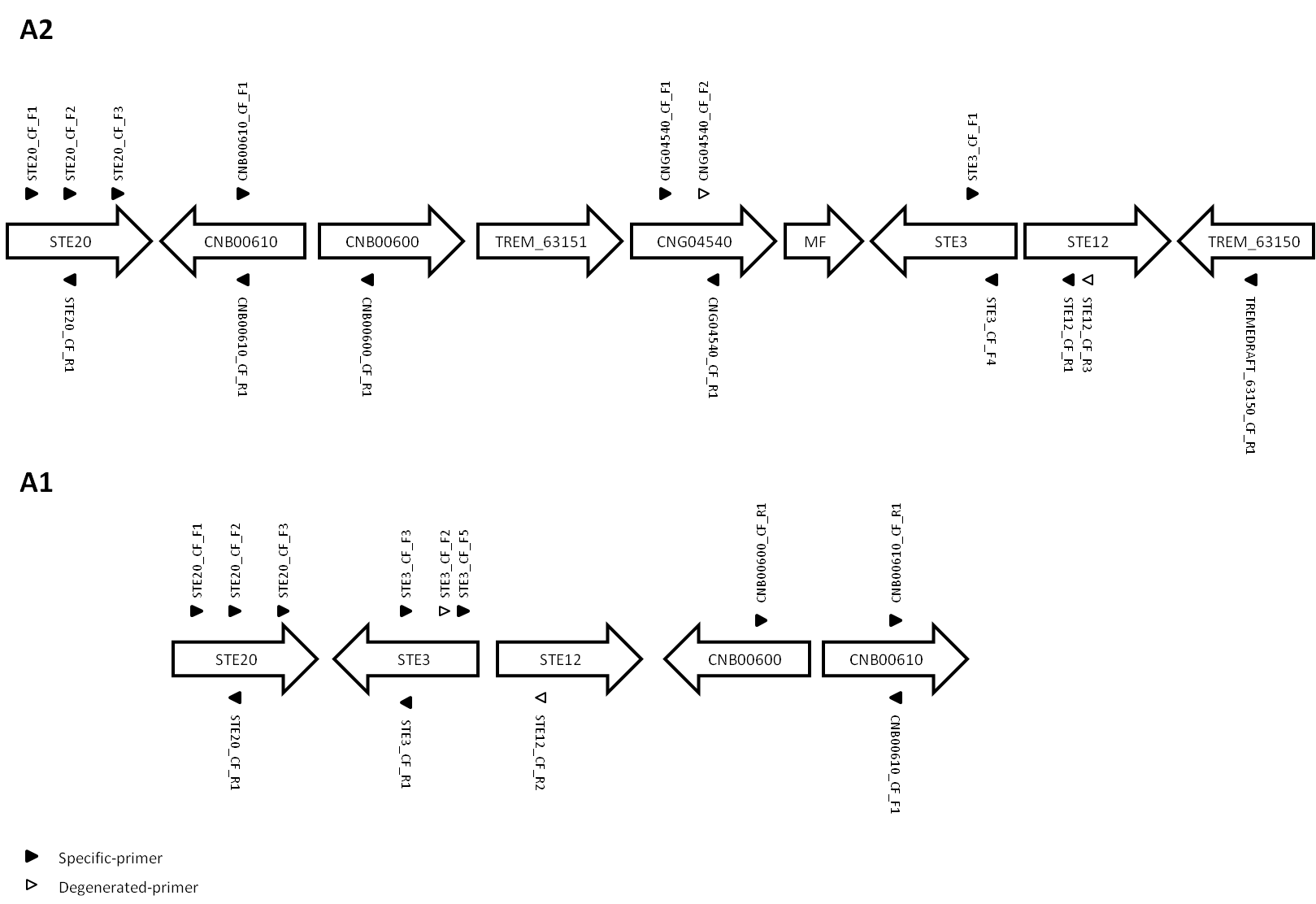

Supplement: S1 File — (DOCX) [file pone.0120400.s006.docx]
